# Telemedicine Application in the Care of Diabetes Patients: Systematic Review and Meta-Analysis

**DOI:** 10.1371/journal.pone.0079246

**Published:** 2013-11-08

**Authors:** Milena Soriano Marcolino, Junia Xavier Maia, Maria Beatriz Moreira Alkmim, Eric Boersma, Antonio Luiz Ribeiro

**Affiliations:** 1 Medical School, Universidade Federal de Minas Gerais, Belo Horizonte, Brazil; 2 University Hospital, Universidade Federal de Minas Gerais, Belo Horizonte, Brazil; 3 Erasmus MC, Rotterdam, The Netherlands; University of North Carolina at Chapel Hill, United States of America

## Abstract

**Background:**

The impact of telemedicine application on the management of diabetes patients is unclear, as the results are not consistent among different studies. The objective of this study is to conduct a systematic review and meta-analysis of randomized controlled trials (RCTs) assessing the impact of telemedicine interventions on change in hemoglobin A1c (HbA1c), blood pressure, LDL cholesterol (LDL-c) and body mass index (BMI) in diabetes patients.

**Methods:**

Electronic databases MEDLINE, Cochrane Central Register of Controlled Trials and LILACS were searched to identify relevant studies published until April 2012, supplemented by references from the selected articles. Study search and selection were performed by independent reviewers. Of the 6.258 articles retrieved, 13 RCTs (4207 patients) were included. Random effects model was applied to estimate the pooled results.

**Results:**

Telemedicine was associated with a statistically significant and clinically relevant absolute decline in HbA1c level compared to control (mean difference -0.44% [-4.8 mmol/mol] and 95% confidence interval [CI] -0.61 to -0.26% [-6.7 to -2.8 mmol/mol]; p<0.001). LDL-c was reduced in 6.6 mg/dL (95% CI -8.3 to -4.9; p<0.001), but the clinical relevance of this effect can be questioned. No effects of telemedicine strategies were seen on systolic (-1.6 mmHg and 95% CI -7.2 to 4.1) and diastolic blood pressure (-1.1 mmHg and 95% CI -3.0 to 0.8). The 2 studies that assessed the effect on BMI demonstrated a tendency of BMI reduction in favor of telemedicine.

**Conclusions:**

Telemedicine strategies combined to the usual care were associated with improved glycemic control in diabetic patients. No clinical relevant impact was observed on LDL-c and blood pressure, and there was a tendency of BMI reduction in diabetes patients who used telemedicine, but these outcomes should be further explored in future trials.

## Introduction

Diabetes mellitus (DM) has taken epidemic proportions throughout the world and is considered among the most challenging health problems in the 21st century [1]. The disease is associated with a number of health-related complications, high morbidity and mortality rates and thus imposes substantial social and economic burdens worldwide [2].

Challenges in the care of diabetic patients require innovative management strategies to improve glycemic and pressoric control, as these are frequently above the desired goals [3]. The optimal management requires an organized, systematic and coordinated approach [2]. Strong evidence demonstrates beneficial effects of patient monitoring and education, focused on a prominent role of the individual self-care with the support of healthcare professionals [4]. This brings into focus the great advances in telecommunication technology and information technology, which can be exploited to improve diabetes management [5,6]. Telemedicine can be a strategy for closer monitoring and intervention to achieve not only better metabolic control, but also to help in the global care of individuals with multiple chronic illnesses [3,7,8]. 

Over the last decade, several studies have addressed the feasibility and efficacy of telemedicine strategies on the management of diabetes patients [9]. Many studies have proved it to be feasible, but the real impact of this of intervention in general and specific clinical situations is still unknown and poorly documented, as the results are not consistent among different studies [6,10]. If proven beneficial, the intervention could be widely disseminated to clinical practice and might help to reduce the burden of the disease. 

The objective of this study is to conduct a systematic review and meta-analysis of randomized controlled trials (RCTs) assessing the impact of telemedicine interventions combined with usual care compared to usual care alone on the management of adult patients with DM type 1 or 2, in terms of change in hemoglobin A1c, blood pressure, LDL cholesterol (LDL-c) levels or body mass index (BMI).

## Methods

This systematic review and meta-analysis was conducted in accordance with the Preferred Reporting Items for Systematic Review and Meta-Analyses (PRISMA) statement [11]. The investigators wrote a protocol and registered it with the International Prospective Register of Systematic Reviews (identification number: CRD42012002779) in August 2012 [12]. 

### Data sources and searches

A literature search with no language restriction was performed using MEDLINE (accessed by Pubmed), Cochrane Central Register of Controlled Trials and LILACS databases to identify relevant studies published until April 2012. On Pubmed, combinations of the MESH terms *diabetes*, *medical informatics*, *clinical decision support system*, *computer assisted decision making*, *information systems* and *telemedicine* were used. In other databases, different combinations of the same terms and the keywords *mobile health*, *telehealth*, *telehealthcare*, *health information technology* were used. The search was supplemented by the reference lists of the identified papers. 

### Study selection

This study included RCTs that compared any strategy of telemedicine application in adult care (i.e. patients ≥18 years) with DM type 1 or 2 in which there was a direct or indirect (i.e. via another healthcare provider) personalized feedback from a healthcare practitioner to the patient about the forwarded clinical data, with a control group not using telemedicine. We restricted ourselves to trials that studied outpatients, and that evaluated at least one of the following outcomes: HbA1c, blood pressure, BMI or LDL-c.

The strategies of telemedicine application included in this review were computerized systems for information exchange, video conferencing, and exchange of information via telephone or other mobile devices, short message service, or through the internet.

Exclusion criteria were as follows: (1) gestational diabetes; (2) studies in patients under 18 years; (3) RCT which did not include feedback from a healthcare practitioner to the patient or other healthcare provider about forwarded clinical data; (4) studies with less than 6 months of follow-up; (5) duplicate publications or substudies of included trials. In this case, the publication with longer follow-up was chosen. 

### Data extraction and quality assessment

Titles and abstracts of retrieved articles were independently evaluated by 2 investigators (J.X.M. and M.S.M.). Abstracts that did not provide enough information for analysis of the intervention or the methodology regarding the inclusion and exclusion criteria defined in this review were retrieved for full-text evaluation.

Subsequently, the 2 investigators independently evaluated full-text articles and determined study eligibility. Disagreements were solved by consensus, and if disagreements persisted, by a third investigator (A.L.R.). The corresponding authors were contacted as needed to obtain data not included in the published report.

The two investigators independently conducted data extraction, and disagreements were solved by the third investigator. Study quality assessment included adequate sequence generation, allocation concealment, blinding of outcomes assessors, use of intention-to-treat analysis, and description of losses and exclusions [13]. Studies without clear descriptions of an adequate sequence generation, and studies with loss to follow-up greater than 20% if analyzed by intention to treat (ITT) and greater than 5% if not ITT were excluded.

### Data synthesis and analyses

The primary outcome was absolute changes in HbA1c from baseline to follow-up. HbA1c is recognized as a valuable indicator of treatment effectiveness in patients with diabetes [2], because it reflects average glycemia over several months and is strongly correlated with diabetes complications [2,14,15].

Secondary outcomes included the absolute changes of LDL-c, systolic blood pressure (SBP), diastolic blood pressure (DBP) and BMI from baseline to follow-up. They were obtained from original studies or calculated (differences in arithmetic means before and after the intervention) [16]. When deviation of the mean difference was not available, the authors were contacted. In case of no response or no availability of the requested information, variance was estimated by using the reported confidence intervals, reported p-values, or an imputation using the standard deviation at follow-up in both groups to calculate the standard error of difference. One study reported data on two intervention groups and one control group [3]. In meta-analysis, data of these intervention groups were combined and compared to the control group.

A random-effects model was used for pooling the included studies, as clinical heterogeneity was expected. Statistical heterogeneity of the treatment effect among studies was assessed using Cochran Q test, and the inconsistency I^2^ test in which values greater than 50% were considered indicative of high heterogeneity. Between-study heterogeneity was performed using the chi-square statistic. A p-value <0.05 was considered statistically significant. 

Sensitivity and subgroup analyses were pre-specified and performed only for the primary outcome. In sensitivity analyses, the effect size was examined by omitting studies individually and also excluding simultaneously studies with extreme results and those with the biggest losses. In this case, the effect sizes were compared by using z tests.

Subgroup analyses aimed to assess whether there was a difference in results of HbA1c in studies which included exclusively patients with DM type 1 vs. type 2, interventions which included changes in patient prescription through telemedicine vs. the ones which did not, intervention made by physicians vs. by nurses, interventions that lasted for 6 months vs. the ones that lasted at least one year. Although not pre-specified in the protocol, a subgroup analysis regarding the average HbA1c on baseline was performed.

A funnel plot of the trial effect size by its standard error was constructed to assess the possibility of publication bias [17]. The symmetry of the plot was evaluated both visually and formally with Egger’s test. The implications for our results were assessed by the fail-safe N and the trim-and-fill method [18].

Statistical analysis was performed using Comprehensive Meta-Analysis version 2.0.

## Results of the Review

### Description of the studies

**Figure 1:**
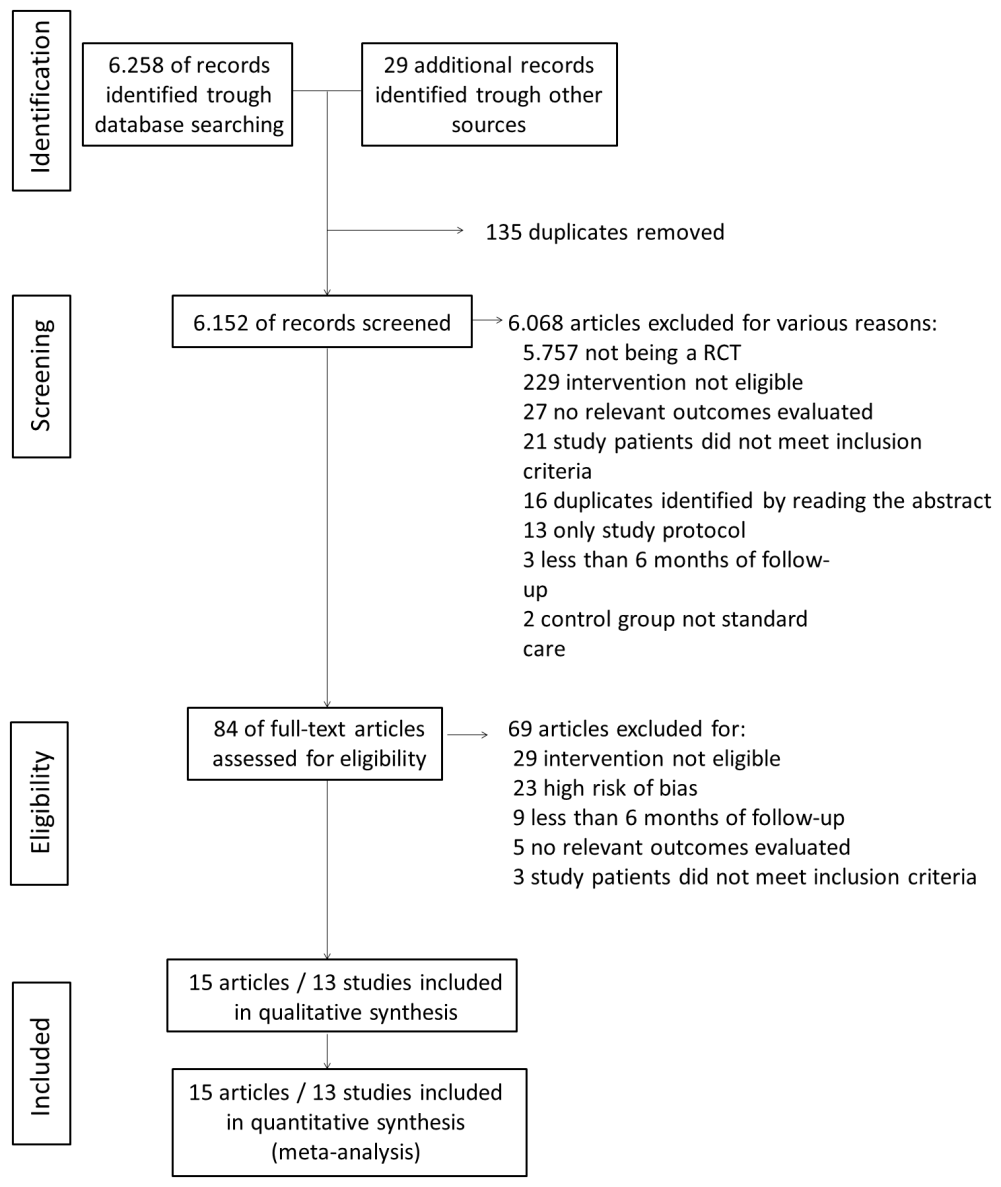
Flow of information through the different phases of the systematic review.

A flow diagram of search and selection is shown in Figure 1. The database search resulted in 6258 (6039 Pubmed, 117 Lilacs, 102 Cochrane Trials) articles and 29 other articles were identified from the references of included studies, totalizing 6287 articles. Of these, 6203 (5984 Pubmed, 117 Lilacs, 102 Cochrane Trials) were excluded after title and/or abstract analysis. Therefore, 84 full text articles were assessed for eligibility. Of these, 69 articles were excluded. Consequently, 15 articles with 13 studies were included in the systematic review. 

The main characteristics of the included studies are summarized in Tables 1 and 2. All 13 studies selected for the review were published in English. Included studies had a total of 4207 patients, 56% were men, 2252 patients were randomized to telemedicine strategies and 1955 to usual care. Ten studies took place in United States and 3 in Europe. One study [19] received funding from industry alone, nine studies [3,10,20-25] were funded by the government alone and three studies [4,26,27] received funding from both. 

**Table 1:** Main characteristics of included studies.

**Study**	**Scenario**	**Mean age in years (SD)**	**Male sex (%)**	**Duration of diabetes I years (SD)**	**Duration of diabetes C years (SD)**	**Mean basal HbA1C (%)**	**Type of diabetes**	**Treatment in use**	**Losses (%)**	**Duration of follow up (months)**	**Outcomes**
**Bond et al, 2007**	University of Washington Diabetes Center, Puget Sound Health System, and local diabetes fairs in the greater Seattle area, United States	67.2 (11.9)	55.0	16.1 (10.5)	17.8 (11.7)	7.1	1 and 2 (87% DM2)	49% insulin, 45% insulin and oral agents and 6% diet and exercise	0	6	HbA1c, BP, weight, total and HDL cholesterol
**Bujnowska et al, 2011**	Primary care units in Poland	55.3 (52.6)	53.5	8.1 (7.6)	7.7 (6.8)	7.6	2	Groups equally divided in insulin users and non-users	5.0	6	HbA1C, BMI, BP
**Charpentier et al, 2011**	Outpatient clinics of 17 hospital sites in France	33.8 (12.9)	36.7	14.7 (9.1)	16.9 (10.5)	9.1	1	Basal-bolus insulin by multiple injections or pump for at least 6 months	10.0	6	HbA1C
**Kirkman et al, 1994 & Weinberger et al, 1995**	General Medicine Clinic of the Durham Department of Veterans Affairs Medical Center, North Carolina, United States	63.5 (19.7)	99.2	11.5 (8.4)	10.3 (7.7)	10.7	2	Currently use of oral hypoglycemic agent or insulin and care at VA general medicine clinic were inclusion criteria	8.7	12	HbA1C
**Montori et al, 2004**	Diabetes clinics Primary care units affiliated with Mayo Clinic, United States	42.9 (4.7)	32.5	19.0 (1.8)	17.9 (2.1)	9.0	1	Multiple daily insulin injections or insulin pumps	19.0	6	HbA1C
**Piette et al, 2000**	2 general medicine clinics of Santa Clara Valley Medical Center in California, United States	55.0 (10.0)	58.5	Not informed	Not informed	8.7	Not informed	Use of a hypoglycemic agent (oral or insulin) was inclusion criteria. 37.5% used insulin	11.0	12	HbA1C
**Piette et al, 2001**	Veterans Affairs outpatients clinics (3 general medicine clinics and 1 diabetes clinic) in California, United States	60.5 (20.0)	97.0	Not informed	Not informed	8.2	Not informed	Active use of a hypoglycemic agent was inclusion criteria. 47% used insulin	6.8	12	HbA1C
**Ralston et al, 2009**	UW Internal Medicine Clinic of Washington University, United States	57.0*	50.6	Not informed	Not informed	8.0	2	Diet, oral medications and or insulin (38% used insulin)	11.0	12	HbA1C, BP, total cholesterol
**Ralston et al, 2009**	UW Internal Medicine Clinic of Washington University, United States	57.0*	50.6	Not informed	Not informed	8.0	2	Diet, oral medications and or insulin (38% used insulin)	11.0	12	HbA1C, BP, total cholesterol
**Rodriguez-Idígoras et al, 2009**	Primary care units in Malaga, Spain	64.0 (21.5)	51.5	11.3 (0.6)	10.2 (0.6)	7.5	2	Diet, oral medications and or insulin	9.5	12	HbA1C, BP, BMI, lipids
**Shea et al, 2006 & Shea et al, 2010**	Primary care units in New York State, United States	71.0 (13.3)	37.0	Not informed	Not informed	7.4	1 and 2	Diet, oral medications and or insulin	14.9	12	HbA1C, BP, lipids
**Smith et al, 2008**	Primary care units affiliated with Mayo Clinic's site in Rochester, United States	61.0 (8.2)	47.5	12.9 (3.3)	14.0 (3.4)	7.3	1 and 2 (93% DM2)	Diet, oral medications (46%) and or insulin (33%)	4.0	21	HbA1C, LDL, systolic and diastolic BP
**Stone et al, 2010**	Veterans Affairs Primary care clinics in Pittsburgh, United States	Not informed	98.6	Not informed	Not informed	9.5	2	Pharmacological treatment for at least 1 year: 76% used oral medications, 58% used insulin	8.6	6	HbA1C, BP, lipids, weight
**Wakefiel et al, 2011**	Veterans Affairs Primary Care Clinics in Iowa City, United States	68.0 (10.0)	98.0	Not informed	Not informed	7.2	2	Not specified	19.0	6	HbA1C, SBP

* Standard deviation was not informed

Abbreviations: BP: blood pressure; DM2: diabetes mellitus type 2; HbA1c: hemoglobin A1c; SD: standard deviation

**Table 2:** Characteristics of included studies regarding the telemedicine intervention.

**Study**	**Intervention**	**Medium used for interaction**	**Target of intervention**	**Applicator of intervention**	**Applicator changed medication**	**Planned frequency of intervention**	**Real frequency of intervention (average)**
**Bond et al, 2007**	Internet program including communication with nurse (SMS, chat, email) and register of self-management activities (glucose, BP) by patients. Nurse monitored data and contacted patients when necessary. Forum discussion with the principal investigator.	Web	Patients	Nurse	No	Not informed, except for the forum discussions: weekly	Not informed
**Bujnowska et al, 2011**	Telehome monitoring system. Patients sent glucose data, and the system analyzed and organized data according to protocols that included alerts and SMS in case of critical values for urgent medical attention.	Telemonitoring device and mobile phone	Patients	Physicians	Yes	Patients should transmit glucose values at least once a week. Practitioners sent feedback in case of critical values	Data transmissions:1.64/patient/week (total 1.850). Interventions (email, phone calls, SMS): 0.42/patient/week (total 474).
**Charpentier et al, 2011**	Diabeo software use plus teleconsultations (group 3). Diabeo: software for patient's smartphones with insulin bolus calculators, plasma glucose targets, automatic algorithms, data transmission to medical staff.	Mobile phone and telephone calls	Patients	Physicians	Yes	Teleconsultations every 2 weeks	8.70 teleconsultations/patient in 6 months. Mean duration of each teleconsultation 7.4 minutes
**Kirkman et al, 1994 & Weinberger et al, 1995**	Calls to educate patients, facilitate adherence to treatment, monitor health status, improve problem solving capacity, and facilitate access to care. Patients could call nurses. Physicians called patients if necessary, according to nurses alerts.	Telephone calls	Patients	Nurse	No	Monthly nurse calls (more frequent if necessary). Patients could call whenever they wanted	13 nurse contacts per patient (1.10/patient/month). 12 min/contact. In 19% of contacts nurses alerted physicians of problems
**Montori et al, 2004**	Transmission of glucose levels and feedback from the study nurse.	Telephone calls	Patients	Nurse*	Yes	Every 2 weeks, 24h after patients transmitted glucometer data	Nurses spent, per patient, 76 min reviewing data, 9 min discussing with endocrinologist and 68 min returning to patients
**Piette et al, 2000**	ATDM: calls in which patients reported clinical information using the keypad and could listen to health promotion automated messages. Nurse reviewed the reports and contacted patients.	Telephone calls	Patients	Nurse	No	Biweekly ADTM calls. Nurse reviewed data weekly and called patients as necessary	1.40 ADTM calls/month. Half of patients completed 78% or more of their attempted assessments
**Piette et al, 2000**	ATDM: calls in which patients reported clinical information using the keypad and could listen to health promotion automated messages. Nurse reviewed reports and contacted patients.	Telephone calls	Patients	Nurse	No	ADTM calls: not informed. Nurse reviewed data weekly and called patients as necessary	15 ADTM calls (1.25/month) and 12 glucose transmissions (1.00/month). Nurse communicated with patients 1.10/month
**Ralston et al, 2009**	Website including electronic medical records, stored glucose values, online diary with feedback, educational website on diabetes. Email exchange with nurses	Web	Patients	Nurses	Yes	Nurse analyzed glucose data at least once a week. Email exchange weekly in the beginning of fw. Patients sent emails to nurses whenever they wanted	76% of patients accessed their medical records, 69% sent emails to nurses, 43% uploaded glucose measures (total 189 uploads) and 33% entered data about diet, exercise, medication
**Rodriguez-Idígoras et al, 2009**	Transmission of glucose values to a call center, which had an alarm and a protocol of interventions in case of abnormal values. Patients could call their physician or the call center staff and vice versa	Mobile phone	Patients	Nurses and physicians	Not informed	Not informed	7.40 glucose transmissions/patient/ month; patients made 3.00 and received 2.60 phone calls per month
**Shea et al, 2006 & Shea et al, 2010**	Home telemedicine unit connected to glucose and blood pressure monitors, videoconferencing, web portal with access to patients clinical data, educational web site created for the project by ADA	Telemonitoring device, videoconferences and Web	Patients	Nurses	No	Not informed	28 videoconferences, 560 glucose uploads, 185 BP uploads, 49 visits to patients web portals, 3 visits to educational site per patient
**Smith et al, 2008**	After a patient visit, an endocrinologist reviewed the records and created a note with suggestions attached to an EB message that was sent to the PCP 48h before the next visit of that patient	Web	Primary care physicians	Physicians (endocrinologists)	No	Each patient visit	Endocrinologists reviewed 1361 consultations, 60% resulted in suggestions. PCP informed they used 49% of them
**Stone et al, 2010**	Telemonitoring device: daily educational reminders, daily transmission of data (glucose, BP, weight). Nurse analyzed and contacted patients when necessary, and also called at least monthly for individualized counseling	Telemonitoring device and telephone calls	Patients	Nurses	Yes*	Daily transmission of glucose and BP. Call whenever necessary, more frequent if high risk detected by the data. Monthly calls for counseling	Duration of nurse-patient telephone contact in hours/patient/month: intervention 1.3h, controls 0.3h. 11% of patients never transmitted data.
**Wakefiel et al, 2011**	Home telehealth device. Patients entered BP and glucose and answered questions, receiving automated feedback. Nurses reviewed data daily and contacted patients by telephone, letter or individualized messages on the device. Physicians contacted if BP or glucose values reached pre-determined values	Telemonitoring device and telephone calls	Patients	Nurses	No	Daily transmissions of data and automated device messages. Nurse and physicians individualized contacts as necessary	Not informed

* Supervised by an endocrinologist

Abbreviations: ADA: American Diabetes Association; ATDM: Automated Telephone Disease Management; BMI: body mass index; EB: evidence-based; PCP: primary care physician; SMS: short message service

Interventions took place in different care settings: 8 studies took place in primary care, 4 studies secondary care and 1 included both primary and secondary care settings. The duration of the intervention was 6 months in 6 studies, 12 months in 6 and 18 months in one study. 

The intervention was particularly targeted at monitoring clinical values, or monitoring combined with education. As per design of our meta-analysis, all studies included personalized feedback from a healthcare practitioner to the patient or other healthcare provider about the forwarded clinical data. The intervention had varying degrees of complexity. Telemedicine strategies included different combinations of transmission of monitoring data, videoconferencing, educational web site, educational reminders, email exchange, evidence-based message to the general physician, teleconsultation, telephone calls, short message service and forum discussion.

Eight trials reported the frequency of data transmission from patients to nurses (six trials) or physicians (two trials). Eight trials provided information about the frequency of feedback of information to patients. The majority of the trials succeeded in data transmission at a maximum frequency of once a week.

### Quality (Risk of Bias) Assessment

Among the included studies, 100% presented adequate sequence generation, 62% reported allocation concealment (8 of 13), 38% had blinded assessment of outcomes (5 of 13), 92% described losses to follow-up and exclusions (12 of 13), and 92% used the intention-to-treat principle for statistical analyses (12 of 13) (Table 3).

**Table 3:** Risk of bias of included studies (n = 13).

**Study**	**Adequate sequence generation**	**Allocation concealment**	**Blinding of outcome**	**Description of losses and exclusions**	**Intention to treat analysis**
Bond et al, 2007	Yes	Unclear	Yes	No*	Yes
Bujnowska et al, 2011	Yes	Unclear	Unclear	Yes	No
Charpentier et al, 2011	Yes	Unclear	No	Yes	Yes
Kirkman et al, 1994 & Weinberger et al, 1995	Yes	Unclear	Yes	Yes	Yes
Montori et al, 2004	Yes	Unclear	Unclear	Yes	Yes
Piette et al, 2000	Yes	Yes	No	Yes	Yes
Piette et al,2001	Yes	Yes	Unclear	Yes	Yes
Ralston et al, 2009	Yes	Yes	Unclear	Yes	Yes
Rodriguez-Idígoras et al, 2009	Yes	Yes	No	Yes	Yes
Shea et al, 2006 & 2010	Yes	Yes	Yes	Yes	Yes
Smith et al, 2008	Yes	Yes	Yes	Yes	Yes
Stone et al, 2010	Yes	Yes	Yes	Yes	Yes
Wakefiel et al, 2011	Yes	Yes	Unclear	Yes	Yes

* The authors were contacted and reported no losses.

### Association of intervention on the primary and secondary outcomes

**Figure 2:**
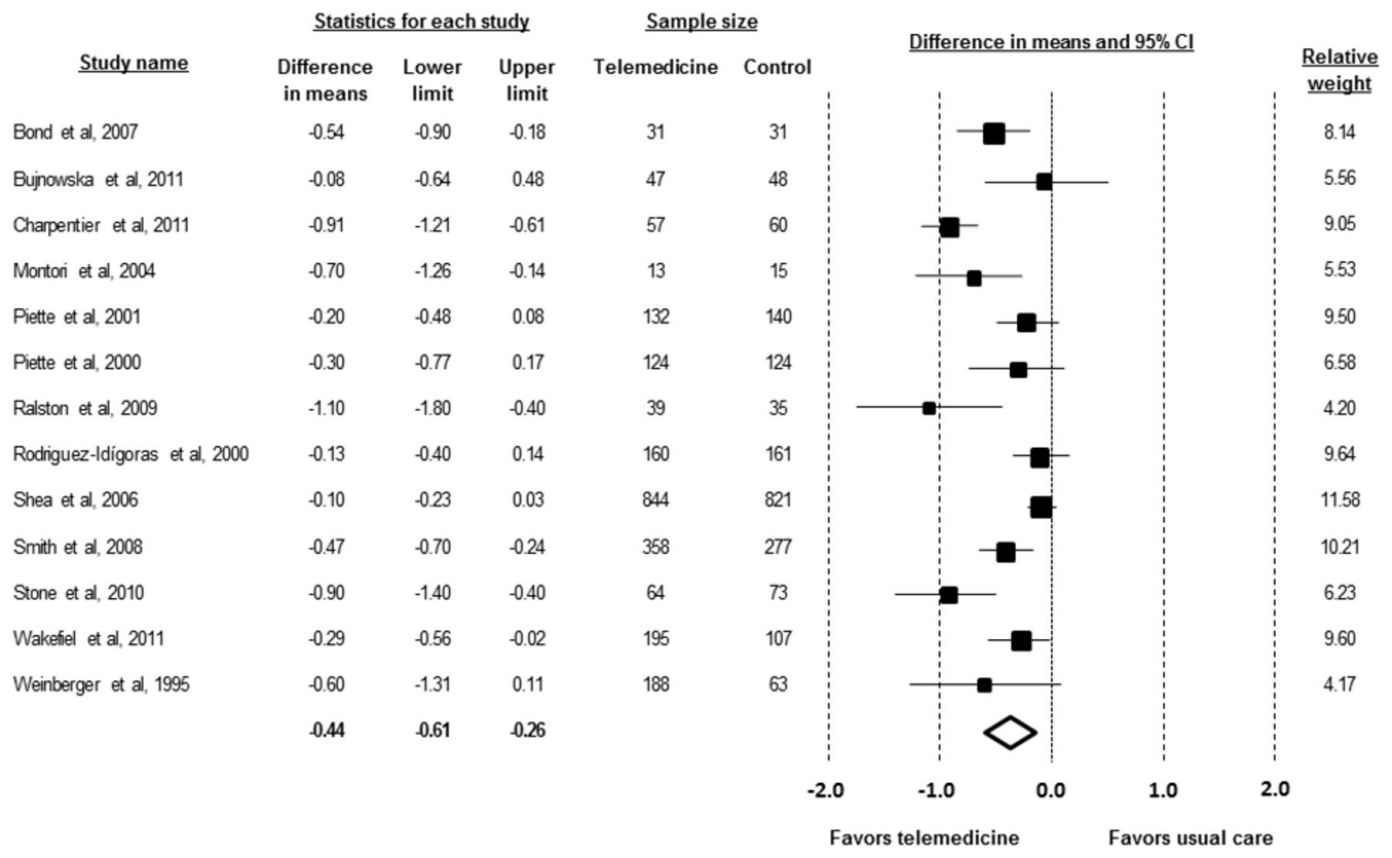
Absolute changes in HbA1c of individual studies of telemedicine associated to usual care vs. usual care.

Telemedicine strategies were associated with a significant absolute HbA1c reduction of -0.44% (-4.8 mmol/mol) (95% confidence interval [CI] -0.61 to -0.26% [-6.7 to -2.8 mmol/mol]; p<0.001) when compared to the usual care (Figure 2). There was evidence of heterogeneity (I^2^ 73%; p for heterogeneity <0.001; range of effects -1.10 to -0.10%). 

**Figure 3:**
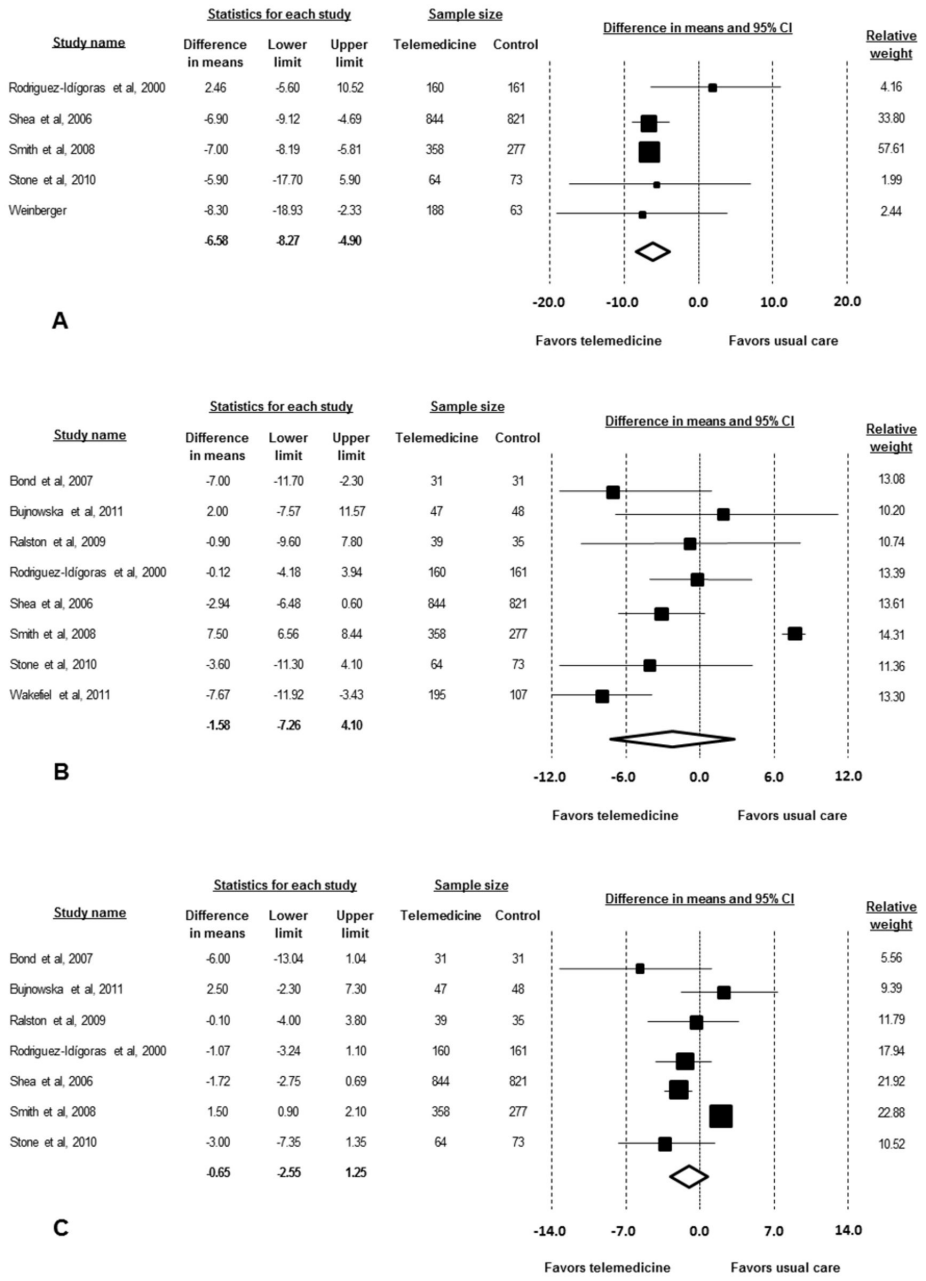
Absolute changes in LDL cholesterol (A), in systolic blood pressure (B) and diastolic blood pressure (C) of individual studies of telemedicine associated to usual care vs. usual care.

Five studies (2889 patients) assessed the effect of telemedicine strategies on LDL-c. There was a significant reduction of 6.6 mg/dL (95% CI -8.3 to -4.9 mg/dL; p<0.001). There was no evidence of a significant heterogeneity (I^2^ 24%; p value for heterogeneity 0.259) (Figure 3A).

Eight studies (3291 patients) assessed the effect on SBP, and 7 (2989 patients) assessed the effect on DBP. There was no significant effect of telemedicine strategies on SBP (effect size -1.6 mmHg; 95% CI -7.2 to 4.1 mmHg; p=0.585) and DBP (effect size -1.1 mmHg; 95% CI -3.0 to 0.8 mmHg; p=0.258) when compared to the usual care (Figures 3B and 3C). There was evidence of heterogeneity for both (SBP I^2^ 94%, p for heterogeneity <0.001, range -7.67 to 7.50 mmHg; DBP I^2^ 84%, p for heterogeneity <0.001, range -6.0 to 1.5 mmHg ).

Only 2 studies (397 patients) assessed the effect on BMI, therefore it was not possible to perform a meta-analysis. Both studies demonstrated a non-significant reduction on BMI.

### Secondary analyses

In sensitivity analyses, studies were individually omitted from the meta-analysis, and the effect size remained fairly the same (Table 4). Even when studies with extreme results and those with the biggest losses [10,22,26] were simultaneously omitted, there was no significant difference in HbA1c reduction (-0.44% vs. -0.56%, p=0.70).

**Table 4:** Sensitivity analyses on hemoglobin A1c (%) effect size removing studies individually.

**Study name**	**Statistics with study removed**		
	**Point**	**Lower limit**	**Upper limit**
Bond et al, 2007	-0,43	-0,62	-0,24
Bujnowska et al, 2011	-0,46	-0,64	-0,27
Charpentier et al, 2011	-0,37	-0,53	-0,21
Montori et al, 2004	-0,42	-0,60	-0,24
Piette et al, 2001	-0,46	-0,66	-0,27
Piette et al, 2000	-0,45	-0,64	-0,26
Ralston et al, 2009	-0,40	-0,58	-0,23
Rodriguez-Idígoras et al, 2000	-0,47	-0,66	-0,28
Shea et al, 2006	-0,48	-0,65	-0,30
Smith et al, 2008	-0,44	-0,63	-0,24
Stone et al, 2010	-0,40	-0,58	-0,23
Wakefiel et al, 2011	-0,46	-0,65	-0,26
Weinberger et al, 1995	-0,43	-0,61	-0,25
**Original effect size**	**-0,44**	**-0,61**	**-0,26**

Subgroup analysis compared studies which included exclusively patients with diabetes type 1 (2 studies, 145 patients) vs. patients with diabetes type 2 (6 studies, 1180 patients) and demonstrated a higher HbA1c reduction in the first group: -0.86% (-9.4 mmol/mol) (95% CI -1.12 to -0.59% [-12.2 to -6.4 mmol/mol]) vs. -0.44% (-4.8mmol/mol) (95% CI -0.74 to -0.15% [-8.1 to -1.6 mmol/mol]), p=0.039.

It was impossible to stratify patients regarding baseline HbA1c because patient-level analyses were not performed. Studies with an average baseline HbA1c equal to or higher than 8.0% [64.0 mmol/mol] were compared to the ones with an average lower than 8.0% and demonstrated a higher impact of telemedicine in the first group: -0.64% (7.0mmol/mol) (95% CI -0.93 to -0.35% [-10.2 to -3.8 mmol/mol]) vs. -0.26% (-2.8 mmol/mol) (-0.43 to -0.10% [-4.7 to -1.1 mmol/mol]), p=0.027.

Interventions which included changes in patient prescription through telemedicine (4 studies, 334 patients) were associated with a more pronounced HbA1c reduction than the ones which did not (7 studies, 3435 patients): -0.75% (-8.2 mmol/mol) (95% CI -1.05 to -0.43% [-11.5 to -4.7 mmo/mol]) vs. -0.30% (-3.3 mmol/mol) (95% CI -0.45 to -0.14% [-4.9 to -1.5 mmol/mol]), p=0.013.

Studies in which the intervention was made by physicians (3 studies, 847 patients) were compared to the ones in which it was made by nurses (9 studies, 3039 patients), and revealed no difference in HbA1c change: -0.53% (-5.8 mmol/mol) (95% CI -0.94 to -0.13% [-10.3 to -1.4 mmol/mol]) vs. -0.43% (-4.7 mmol/mol) (95% CI -0.64 to -0.22% [-7.0 to -2.4 mmol/mol]), p=0.654.

There was a tendency of a higher HbA1c reduction in interventions which lasted 6 months (6 studies, 741 patients) when compared to the ones that lasted at least one year (7 studies, 3466 patients; in only one study the follow-up was longer than one year): -0.57% (-6.2 mmol/mol) (95% CI -0.85 to -0.30% [-9.3 to -3.3 mmol/mol]) vs. -0.30% (-3.3 mmol/mol) (-0.48 to -0.11% [-5.2 to -1.2 mmol/mol]), p=0.099.

### Publication Bias Assessment

**Figure 4:**
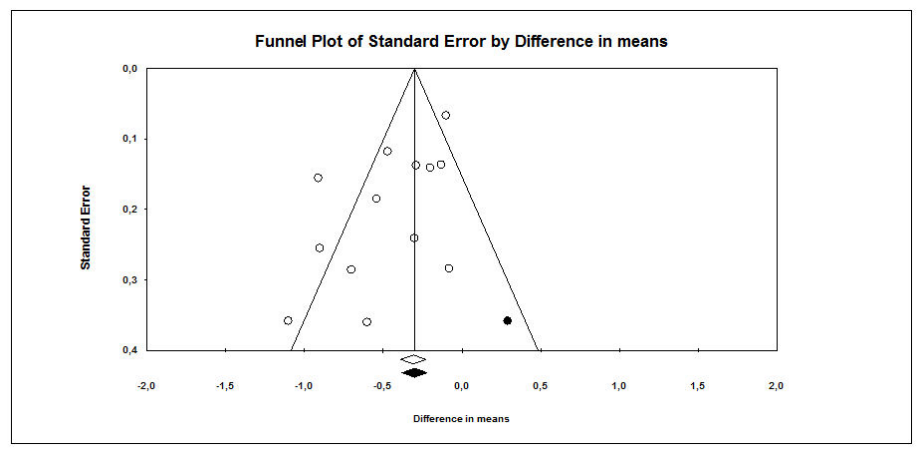
Observed studies are shown as open circles, and the observed point estimate is shown as open diamond. The imputed study is shown as a filled circle, and the imputed point estimate is shown as a filled diamond.

The subject impression of the funnel plot test suggested an asymmetry (Figure 2). Toward the bottom of the graph there are more studies on the left (indication a higher impact), which is consistent with the possibility that some studies are missing from the right. This visual impression was confirmed by Egger’s test, yielding a significant p-value (0.022). However, the fail-safe N for our pooled analysis is 273000, which is reassuring since it is very unlikely that there are over 1000 unpublished or undiscovered studies for every 1 study we found. Additionally, the trim-and-fill method revealed that publication bias did not interfere with the interpretation of results The method suggested that one study is missing, and the imputed effect size was -0.41% (95% CI -0.58 to -0.23%), which is similar to the original effect, suggesting that the apparent publication bias is insufficient to affect our results or interpretations in a meaningful way (Figure 4).

## Discussion

This systematic review (13 studies, 4207 patients) indicates that in diabetes patients, telemedicine strategies concomitant to the usual care are associated with a mean HbA1c decline of −0.44% (-4.8 mmol/mol) when compared to the usual care alone. HbA1c is recognized as a valuable indicator of treatment effectiveness in patients with diabetes [2], because it reflects average glycemia over several months and is strongly correlated with diabetes complications [2,14,15]. Additionally, our analyses demonstrate that there was no clinical relevant impact on LDL-c (an effect size of only 6.6 mg/dL) and no impact on blood pressure. It was impossible to perform a meta-analysis to assess the impact on BMI, but there is a tendency of its reduction with the use of telemedicine.

The lack of a clinical significant impact on LDL-c and the absence of impact on blood pressure should not be interpreted as a definite finding. Regarding hypertension, a recent RCT which included a large sample of hypertensive patients (7.3% of them had diabetes) who were willing to monitor their blood pressure and self-titrate medication has shown that self-management of hypertension in combination with telemonitoring of blood pressure measurements resulted in significant reduction in blood pressure in 6 and 12-month follow-up when compared to usual care [27]. No study in the present meta-analysis included an intervention similar to that one. Therefore, the telemedicine strategy design might have not been appropriate to generate a significant impact on hypertension and LDL-c, and the intervention might be more effective in a motivated sample of patients, as was the case. This should be in mind when designing new studies to address these outcomes.

Our findings also demonstrated that the impact of telemedicine strategies in reducing HbA1c was more pronounced in diabetes type 1. Type 1 diabetes patients are unique in that they have absolute insulin deficiency and consequently the have more pronounced glycemic variability than type 2 diabetes patients [2,28]. Their management always involves insulin administration, whereas type 2 diabetes patients may be managed using lifestyle changes and oral hypoglycemic agents. These differences could influence the impact of individual’s self-management strategies and affect outcome measures [29]. Additionally, type 2 diabetes is more common in older adults. Compared with younger adults, older adults report greater anxiety about using computers, lower use of technology and less confidence in their technological abilities [6] Therefore, some patients may have found difficulties in dealing with the telemedicine applications, for example, to upload blood glucose results to the website or to access online educational materials.

Interventions which included changes in patient prescription through telemedicine were associated with a greater benefit on HbA1c reduction than the ones which did not, and there was no difference regarding intervention made by physicians or nurses. These findings are important to address recommendations on telemedicine design strategies in clinical practice. The lack of difference between physicians and nurses impacts significantly in decreasing the costs of the intervention if applied to the healthcare system.

Another important finding was the tendency of a higher HbA1c reduction in interventions which lasted 6 months when compared to the ones that lasted at least one year. Although it did not reach statistical significance, it suggests a trend of decreasing intervention impact over time. This finding suggests that in clinical practice contact through telemedicine and positive motivation should be intensified over time, in order not to decrease the impact on glycemic control. Additionally, a more participatory design approach, involving target users in developing personalized interventions, might enhance persistence in usage and adherence to treatment [30].

This study was a rigorous systematic literature review that focused exclusively on RCTs in which telemedicine strategies included a personalized feedback from a healthcare practitioner to the patient or to other healthcare provider. Although only 5 studies had blinded assessment of outcomes, the outcomes were objective measures, with a low risk of bias in their assessment.

Previous systematic reviews with less methodological rigor have not performed meta-analysis or failed to detect a significant impact of different telemedicine strategies on glycemic control [6,26,30-32]. Methodological limitations may have biased the results, including inaccurate or incomplete indexing in the database, or a weak search strategy, leading to incomplete accounting of existing studies; limited or no assessment of study quality; and inclusion of weak studies. One systematic review demonstrated a significant decrease in HbA1c, but with a lower impact (effect size -0.22% [-2.4 mmol/mol]; 95% CI -0.35 to -0.08% [-3.8 to -0.9 mmol/mol]) [33]. The inclusion of RCTs independent of study quality and the possibility of selection bias on observational studies may have influenced the results. 

The evidence presented here could support the implementation of telemedicine in the care of diabetes patients. It has already been shown that telemedicine help improving patient care and access to specialized care, in particular in rural areas with limited accesses to health care facilities [34], and it is expected to facilitate a productive interaction between the patient and the health care provider in order to achieve improved treatment results and lower treatment costs [35]. This review did not address the cost-effectiveness of the intervention. Another systematic review, specific on teleconsultation, reported controversy about that, but cost-effectiveness was not measured uniformly among the various studies, and some of them were observational studies without a control group [30]. This question is of utmost importance for decision makers and therefore should be addressed in further clinical trials.

In order to make these results applicable to clinical practice, it is also important to take into account that telemedicine may not be appropriate for all patients. There are some factors that are described as important elements for the success, such as awareness of the significance of the disease and its consequences, willing to participate actively in the treatment and in preventing complications and positive motivation [4]. Patient computer skills and adherence to the technology are also important depending on the complexity of the telemedicine strategy [36].

The main limitation of this meta-analysis is the heterogeneity. To address this, we have performed analyses to identify clinical and methodological differences between studies. Some factors could not be addressed, as the type of telemedicine intervention, because it was too diverse among studies. As the analyses were not based on individual data, it was not possible to stratify according to the basal HbA1c, and it is expected a higher impact in individuals with poor metabolic control. The inclusion of patients with good metabolic control may have underestimated the impact of the intervention, so it could be expected an even better result if we could stratify by the baseline HbA1c levels. The subgroup analysis comparing studies with an average HA1c lower or higher than 8.0% (64.0 mmol/mol) is limited, because it is not possible to effectively separate individuals with better and worst metabolic control. However, as it demonstrated a significantly higher impact on HbA1c in the subgroup of studies with baseline HbA1c equal to or higher than 8.0%, it provides an estimative of a higher impact of the intervention in patients with worst metabolic control.

Although there was evidence of publication bias, trim-and-fill method revealed that publication bias did not interfere with the interpretation of results, as the imputed effect size was similar to the original effect, suggesting that the apparent publication bias is insufficient to affect our results or interpretations in a meaningful way.

It is important to note that the endpoint HbA1c has some limitations. As a mean, it may show normal results in individuals who alternate hypo and hyperglycemias [37]. Besides this, it is necessary that laboratories use HbA1C assay methods certified by the National Glycohemoglobin Standardization Program (NGSP) as traceable to the DCCT (Diabetes Control and Complications Trial) reference [38,39]. Finally, some clinical situations can lead to a HbA1C falsely high or falsely low, such as pregnancy, hemoglobinopathies, disorders of erythrocyte turnover, renal failure [37,40].

## Conclusions

In conclusion, this systematic review and meta-analysis has shown that telemedicine strategies combined with the usual care are associated with improved glycemic control in diabetic patients. In this review, there was no clinical relevant impact on LDL-c and blood pressure, and there was a tendency of BMI reduction in diabetes patients who used telemedicine, but the impact on these outcomes should be further explored in future trials.

A logical next step for future trials would thus be to assess whether the improved glycemic control observed with telemedicine interventions will translate into improved clinical outcomes. The optimal design of telemedicine systems is uncertain, so future research should also focus on what type of telemedicine intervention has the largest impact on diabetes management, and how to adapt systems to the individual patient needs and resources of specific health care systems. The evaluation of the cost-effectiveness of the intervention should also be addressed, which is relevant to decision makers and impacts on the applicability of the evidence in clinical practice. 

## Supporting Information

Checklist S1 **PRISMA checklist.**
(DOC)
